# Equity and the Sun Quality Health Private Provider Social Franchise: comparative analysis of patient survey data and a nationally representative TB prevalence survey

**DOI:** 10.1186/1475-9276-12-5

**Published:** 2013-01-10

**Authors:** Dominic Montagu, May Sudhinaraset, Thandar Lwin, Ikushi Onozaki, Zaw Win, Tin Aung

**Affiliations:** 1 Global Health Group, University of California San Francisco, San Francisco, CA, USA; 2 National TB Program, Myanmar Ministry of Health, Naypyidaw, Myanmar; 3 StopTB Department, WHO, Geneva, Switzerland; 4 Population Services International, Yangon, Myanmar

**Keywords:** Tuberculosis, Private providers, Myanmar, Poor, Urban rural

## Abstract

**Introduction:**

Since 2004, the Sun Quality Health (SQH) franchise network has provided TB care in Myanmar through a network of established private medical clinics. This study compares the wealth distribution of the TB patients to non-TB patients to determine if TB is most common among the poor, and compares the wealth of all TB patients to SQH TB patients to assess whether the franchise achieves its goal of serving the poor.

**Methods:**

The study uses data from two sources: 1) Myanmar’s first nationally representative TB prevalence study conducted in 2009, and 2) client exit interviews from TB patients from SQH clinics. In total, 1,114 TB-positive individuals were included in the study, including 739 from the national sample and 375 from the SQH sample.

**Results:**

TB patients at SQH clinics were poorer than TB-positive individuals in the overall population, though not at a statistically significant level (p > 0.05). After stratification we found that in urban areas, TB patients at SQH clinics were more likely to be in the poorest quartile compared to general TB positive population (16.8% vs. 8.6%, respectively; p < 0.05). In rural areas, there was no statistically significant difference between the wealth distribution of SQH clinic patients and general TB positive individuals (p > 0.05).

**Conclusion:**

Franchised clinics in Myanmar are reaching poor populations of TB patients in urban areas; more efforts are needed in order to reach the most vulnerable in rural areas.

## Introduction

Access to quality tuberculosis (TB) treatment is increasing globally both in absolute and relative numbers. The World Health Organization (WHO) reports that 65% of the 8.7 million incident cases of TB were reported in 2010. The 2009 treatment success rate, the percentage of new, registered, smear-positive (infectious) cases that were cured or in which a full course of treatment was completed, was 86%. Despite this TB remains a significant cause of illness, resulting in 2.5 millions deaths in 2010 [1]. TB remains a disease of the poor around the world, and the economic effects of illness can be catastrophic to low-income families [2,3].

Part of the reason for the high rates of untreated TB in many low- and middle-income countries is that while national TB programs commonly provide effective and free diagnosis and treatment the majority of the population does not seek medical care in government facilities [4]. The high cost of transport to government facilities and waiting times for care once there drive poorer patients to seek treatment in private settings despite higher costs of medicines and often- uncertain quality [5,6]. Delays in treatment due to mis-diagnosis and inappropriate treatment in private settings are widespread [7-9]. Addressing the barriers faced by the poor in being treated for TB therefore requires assuring quality of care while at the same time providing accessible close-to-client care, and minimizing the direct cost of treatment.

Since 2000, WHO and the STOP TB Partnership have worked to address these issues by supporting governments in the 22 designated ‘high-burden countries’ [1] to improve and integrate private care into national TB program strategies using a combination of methods summarized as public-private-mix-directly-observed-therapy (PPM-DOTS) [4,10]. The efficacy of PPM-DOTS programs is measured against the four global objectives of the STOP TB Partnership: to 1/ increase TB detection rates, 2/ improve TB treatment outcomes, 3/ enhance access and equity, and 4/ to reduce financial burden upon patients.

The data for the first and second of these outcomes indicates success: PPM-DOTS programs have been effective at increasing case detection rates, and have achieved treatment completion rates that are better than non-PPM private provision, and equal or better to private DOTS initiatives in high burden countries [11,12]. What this aggregate evidence fails to show, is whether or not the benefits of these programs are shared equitably. A recent systematic review found little evidence to indicate whether or not PPM-DOTs programs increase equity by reaching patients who were poorer, or with less access to services, than those who would receive care from traditional care sources [13]. This study attempts to fill this gap by examining an established PPM-DOTS initiative in Myanmar to assess the extent to which it serves the poor and disadvantaged.

### Context

Myanmar is one of the poorest countries in Asia, with low overall spending on health, and an estimated TB prevalence rate (525/100,000), among the highest in the region [1]. The government has a well-managed TB treatment program with 85% treatment success rate, however it only identifies an estimated 70% of all new cases.^a^ Geographic access to government care limits utilization of national program centers, and there is a patient preference for private treatment, which is viewed as both more accessible, and of higher quality than government services [14]. In response to this a number of initiatives have been created to engage private practitioners in TB identification, referral, and treatment [12]. Among the largest is an initiative of the US-based NGO, Population Services International (PSI). PSI began working in Myanmar in 1995 and since 2003 has supported a growing number of private doctors operating branded social franchises to diagnose and treat tuberculosis [15].

### Social franchising

Social franchising is a model for applying the contracting and managerial systems of commercial service franchising to social aims [16]. Having evolved from commodity social marketing programs in Asia in the early 1990s [17], social franchising is now a well established method for delivering subsidized health services to large numbers of people in low- and middle-income countries around the world [18]. While the evidence on overall effectiveness of social marketing programs remains limited [19], recent studies have provided indications of of improvements in access, quality, and patient and provider satisfaction using this social franchising delivery model [20-24]. A recent systematic review concluded that the evidence for impact is positive, but weak, with most studies included scoring between 2 and 4 (out of a possible 9 in a WHO-Johns Hopkins rigour scale) in terms of the strength of their study designs (Beyeler et al. unpublished).

Most social franchise programs have focused on supporting the delivery of family planning services; however there is a growing trend towards diversification [25]. In 2007 Lonnroth et al. used archival reporting data and pre-post intervention data to demonstrated the increase in both TB diagnosis and treatments resulting from the introduction of franchised services in Yangon, and the effectiveness of the program at reaching lower income populations [26]. That study was limited, however, by the focus on only one city, by use of a non-representative metric for socio-economic status, and by the small sample size and uncertain frame for national reporting data used.

### Study goals

This study uses national data on household assets and TB prevalence to determine the equity of the SQH programs. By determining national wealth estimates, and comparing both urban and rural populations to TB patients treated in SQH clinics we sought to verify the findings of Lonnroth et al., extending the equity analysis more broadly to all urban and rural areas of Myanmar, and to compare the wealth distribution of SQH-treated TB patients both to the overall population, and to the population of TB-positives across the country. Our hypotheses were that 1) TB-positive individuals would be poorer than the overall population; and 2) the SQH-treated TB patients would be poorer than the overall TB-positive population.

## Methods

### Data sources

The study analyzes existing data from two existing sources. First, data obtained from 3 rounds of PSI/Myanmar client follow-up interviews conducted with TB patients of Sun Quality Health (SQH) clinics across the country. The first round was carried out in March-April 2010. From a frame of 1228 TB patients registered with SQH clinics in December 2009 we randomly selected 123 (10% sample). A second round of follow-up interviews were carried out in August-September 2010 based on 1336 patients registered in June 2010, again using a 10% random sample (134 records), and a third round was carried out in March 2011 based on patients from December 2010 (1184 patients for the month; random 10% sample of 118 records). To select each sample all patients were sorted by registration date and the alphabetic order of the clinicians where registration occurred. Every 10th was then selected using a random start. Contact information for these patients was determined from the SQH clinics where they registered and received treatment. Research staff contacted patients at their residence and after receiving consent, conducted in person interviews. Because of incomplete address information 64 selected patients (17.1%) were not found. These were replaced using the subsequent listed name from the sampling frame. Only one patient (0.3%) refused to be interviewed. Altogether 375 TB patients successfully completed the interviews.

Second, we used data from Myanmar’s first nationally-representative TB prevalence survey conducted in 2009 through the National TB Programme (NTP). The goal of the survey was to measure the prevalence of TB in Myanmar, using a nationally-representative sample, based on a stratified multi-stage household cluster sample. Stratification was conducted on states and divisions, equivalent terms for the highest level of political divisions within Myanmar. Countrywide, 70 out of 293 townships were sampled. At each selected household biological specimens (sputum samples) to test for TB were collected and a questionnaire was administered to all adult residents. In total, data was collected from 51,367 participants, aged 15 years and older. Among these, 739 participants were classified as having “active TB” based on sputum test results confirmed by chest x-ray reviewed independently by two radiologists.

We conducted comparative analyses of the active-TB subgroup together with the SQH patients. Combining these two data sources, the analytic sample included 1,114 individuals, with 739 participants from the national survey and 375 participants from the clinic survey.

### Variables of interest

After combining the two datasets, we constructed a participant type variable in order to distinguish participants from the SQH exit interview surveys and NTP survey. Demographic variables of interest included rural/urban residence, gender, age (categorical variable), and education level (categorical variable including illiterate, primary, secondary, and college).

We created an index of socioeconomic status using a set of household asset variables and principal components analysis (PCA). PCA is a statistical tool used to determine the orthogonal linear combinations of variables that capture shared or common information most efficiently; in other words, instead of assigning an arbitrary weight to each variable, or assigning equal weights to each asset variable, it allows us to determine variables that maximize the explained variance to measure an underlying index (i.e. socioeconomic status) [27,28]. In this analysis, the wealth index used a list of 10 asset variables that were included in both the NTP survey and SQH client exit surveys. We first combined the datasets, and then conducted PCA on the entire sample. We then conducted PCA stratified analyses for rural and urban samples separately, as recommended by existing literature [29]. A continuous wealth index was constructed, and then further categorized in to wealth quartiles. Graphical distributions of categorization of wealth indices were compared, as well as distributions by sample and percentage of households in each wealth category.

### Analysis

All analyses were performed using Stata version 12MP. Descriptive analyses consisted of tabulating means, assessing missing values of key variables, standard errors, and frequency distributions. We used chi-square statistics and assessed p-values to determine differences between the two groups (SQH clinic sample vs. general population sample) and wealth quartiles. We also stratified analyses by rural and urban residence.

This study was conducted in accordance with established ethical standards and exempted from review by the UCSF Committee on Human Research.

## Results

**Figure 1:**
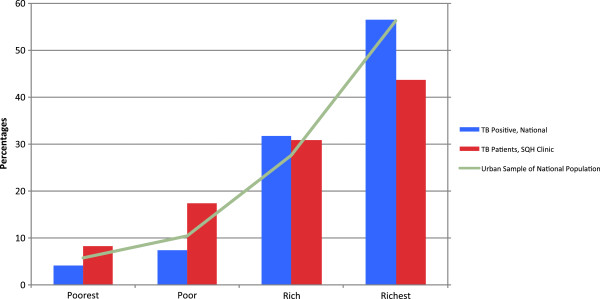
Wealth Distribution of Overall Population, TB positives, and SQH TB patients (Urban).

**Figure 2:**
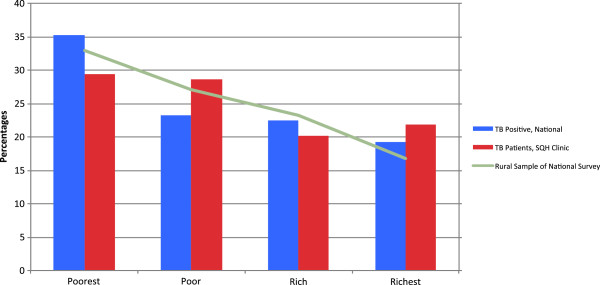
Wealth Distribution of Overall Population, TB positives, and SQH TB patients (Rural).

Demographic statistics are shown in Table 1. As is common around the world, the urban population of Myanmar is significantly better off than the rural population: 55% of all urban residents are in the richest wealth quartile (Figure 1). The rural population is correspondingly less wealthy, with 33% of rural residents in the poorest quartile (Figure 2). Within the national survey data, active-TB is not associated with poverty after stratification by urban/rural residence (p > 0.05).

**Table 1:** Demographic characteristics of TB positive study participants, by source

	**National TB Participant** ** % (n = 739)**	**SQH Clinic Participant % (n = 375)**	**Total % (N = 1114)**	**Chi2, P-Value**
Urban	25.4	52.3	34.4	80.0, 0.000
Rural	74.6	47.7	65.6	
**Age Group**				
15-24	5.1	38.1	16.2	231.5, 0.000
25-34	13.4	17.6	14.8	
35-44	18.8	15.2	17.6	
45-54	22.7	12	19.1	
55-64	17.4	9.9	14.9	
65+	22.6	7.2	17.4	
**Gender**				2.9, 0.087
Male	64.7	59.5	62.9	
**Highest Education Level**				50.8, 0.000
Illiterate	31.5	15.9	27.6	
Primary	34.6	27.8	32.9	
Secondary	29.4	43.7	33	
College/Grad	4.4	12.7	6.5	

The SQH network serves a predominantly urban population; indicative of the urban concentration of private doctors in Myanmar. Quintile-level comparisons show that within urban areas, SQH clinics are pro-poor, serving a higher percentage of poor patients than that found in the wealth distribution of nationally representative active-TB individuals. Twenty-five percent of SQH TB patients are in the lowest wealth quintile, compared with 16.6% of national sample (Table 2). Similarly, in urban areas the national sample has a greater proportion in the highest wealth quintile than patients in SQH clinics (21.9% vs. 8.7%). Both ends of the wealth comparison were statistically significant (p < 0.05).

**Table 2:** Distribution of wealth quintiles, by sample and rural/urban residence

	**Rural**				**Urban**			
	**National**	**SQH**	**Total**	**Chi-2, p-value**	**National**	**SQH**	**Total**	**Chi-2, p-value**
Poorest	30.8%	32.4%	31.2%	11.47,p = 0.022	16.6%	25.5%	21.1%	15.69, p = 0.003
Poor	13.2%	4.5%	11.1%		18.7%	19.9%	19.3%	
Average	17.4%	20.1%	18.1%		20.3%	18.9%	19.6%	
Rich	19.7%	19.6%	19.7%		22.5%	27%	24.8%	
Richest	18.8%	23.5%	20%		21.9%	8.7%	15.1%	

In rural areas, a greater proportion of the TB positive individuals identified in the national sample reported being in the lowest two quintiles when compared to SQH clients. Differences across the two groups were small (44.0% vs. 36.9%, summing poorest two quintiles), but statistically significant (p = 0.02) (Table 2).

We confirmed these findings using quartile divisions of wealth: a stronger possible analysis in light of the limited number of household assets available in the national survey. Only weak statistical differences were found in wealth distributions between national vs. SQH samples (p = 0.058) (Table 3). In urban areas, however, SQH clients were more likely to fall in the poorest and poor categories compared to the national sample (56.1% vs. 48.7%, summing poorest two quartiles, Table 3). Graphical representation of the urban – rural division between overall population wealth distribution, TB incidence, and SQH TB patients, is shown in Figures 1 and 2.

**Table 3:** Distribution of wealth quartiles, by sample and rural/urban residence

	**Rural**				**Urban**			
	**National**	**SQH**	**Total**	**Chi-2, p-value**	**National**	**SQH**	**Total**	**Chi-2, p-value**
Poorest	30.8%	32.4%	31.2%	7.47, p = 0.058	27.3%	35.2%	31.3%	13,50, p = 0.004
Poor	20.1%	15.6%	19%		21.4%	20.9%	21.1%	
Rich	27.5%	21.8%	26.1%		18.7%	26.5%	22.7%	
Richest	21.6%	30.2%	23.7%		32.6%	17.3%	24.8%	

## Discussion and conclusions

Our analysis of national TB prevalence data and patient data from SQH private providers provides both an overview of the wealth distribution among TB infected individuals within Myanmar, and provides a basis to test the extent to which the social franchise program supported by PSI/Myanmar for the delivery of TB treatment achieves its goal of serving the poor.

This national TB prevalence data found that 79.3% of the total Myanmar population resides in rural areas [data not shown], and that 74.6% of all TB cases occur in this population [Table 1]. The rural/urban divide was statistically significant (p < .001). After differentiating between urban and rural populations, however, there is no statistical basis to argue that, in Myanmar, TB is a disease of the poor. TB levels in each wealth quartile approximate the national prevalence rate of 1,400/100,000.

Private treatment through the SQH social franchise program is less evenly distributed. In rural areas the SQH franchise providers are caring for patients that are not statistically different in wealth than the general population of TB-infected individuals. In urban areas, SQH clinics are treating patients that are poorer than the general infected population. These findings suggest that franchises are successfully reaching low-income TB patients in urban areas, but could improve targeting of lower socioeconomic groups in rural areas.

Our study suffers from some limitations. First, TB diagnosis was conducted using assessment measures (x-ray scans), which are standard in Myanmar but no longer considered appropriate in many other countries. Clinic data from SQH facilities included those in treatment- only and therefore is biased toward treatment-seeking individuals with a resultant number of possible confounding or mediating attributes such as education level, social capital, and migration experiences - to name only a few – for which we lacked measures and may have missed as a result. For these reasons, the SQH patient sample is not a perfect match for the national prevalence survey in which only 0.16% of those identified with active TB were undergoing treatment at the time of the survey. The national survey also suffers from selection bias: inclusion criteria restricted the survey to individuals 15 and older, and therefore pediatric TB cases are not represented. Because our study was conducted through analysis of existing secondary data, a-priori power calculations were not possible.

Despite these constraints our study is a contribution to the field as it provides the strongest evidence yet that private providers organized and supported through a social franchising network are able to effectively target, and serve the poor. The pro-poor aims of social franchises around the world have often been called into question because of the contradictions inherent in providing care through a clinic network incentivized by fee-charging providers. Our study has confirmed the effectiveness of the SQH social franchise’s pro-poor intentions.

We would advise that the analysis we have conducted here be standardized in social franchise and other healthcare service delivery programs around the world through the incorporation of a standardized asset measure questionnaire given to patients served and matching assets used in DHS or other nationally representative surveys.

## Endnote

^a^Based on 2010 case notification and treatment data [1].

## Competing interests

MZ and TA work for Population Services International in Myanmar. The authors declare no other competing interests.

## Authors’ contributions

DM and TA conceived of the study comparison. DM led the writing and interpretation of results. MS carried out the final data analysis and contributed to the writing. TL and IO led the analysis of national survey data. ZW led the analysis of SQH data. All authors gave input to the paper conclusion. All authors read and approved the final manuscript.
